# Co-infections with multiple pathogens in natural populations of *Ixodes persulcatus* ticks in Mongolia

**DOI:** 10.1186/s13071-022-05356-x

**Published:** 2022-06-28

**Authors:** Ekaterina K. Lagunova, Natalia A. Liapunova, Davaakhu Tuul, Gerechuluun Otgonsuren, Davaadorj Nomin, Nyamdorj Erdenebat, Davaajav Abmed, Galina A. Danchinova, Kozue Sato, Hiroki Kawabata, Maxim A. Khasnatinov

**Affiliations:** 1 Laboratory of Vector-Borne Infections, FSPSI Scientific Centre for Family Health and Human Reproduction Problems (SC FHHRP), Irkutsk, Russian Federation; 2 Selengie Aimag Department, National Center For Zoonotic Diseases, Sukhbaatar, Mongolia; 3 National Centre For Communicable Diseases, Ulaanbaatar, Mongolia; 4 Department of Bacteriology-I, National Institute of Infectious Diseases, Shinjuku-ku, Tokyo, Japan

**Keywords:** Mongolia, *Borrelia burgdorferi* sensu lato, *Borrelia miyamotoi*, Tick-borne encephalitis virus, *Anaplasma phagocytophilum*, *Ehrlichia* sp*.*, *Riskettsia sibirica*, *Rickettsia heilongjiangensis*, *Ixodes persulcatus*, Co-infection

## Abstract

**Background:**

In Mongolia, the taiga tick *Ixodes persulcatus* is the major vector of tick-borne pathogens. Knowledge about co-infections of these pathogens in ticks is necessary both for understanding their persistence in nature and for diagnosing and treating tick-borne diseases.

**Methods:**

The prevalence of seven tick-borne infections in 346 *I. persulcatus* collected from the Selenge and Bulgan provinces of Mongolia was evaluated using real-time PCR. Quantification of *Borrelia* spp. was performed using multiplex quantitative PCR targeting the* 16S* rRNA gene. Genetic analysis of *Borrelia* spp. in 11 ticks infected with *Borrelia miyamotoi*, including six ticks co-infected with *Borrelia burgdorferi* sensu lato (s.l.), was performed by high-throughput sequencing of the *flaB* gene fragment.

**Results:**

Six ticks (1.7%) were infected with tick-borne encephalitis virus (TBEV); 171 (49.4%), with *B. burgdorferi* sensu lato; 17 (4.9%), with *B. miyamotoi*; 47 (13.6%), with *Anaplasma phagocytophilum;* and 56 (16.2%), with *Ehrlichia* sp. Neither *Rickettsia sibirica* nor *R. heilongjiangensis* were detected. *Borrelia burgdorferi* s.l. occurred as co-infection in 55 (32.2%) of all infected ticks. The other pathogens co-infected ticks in 58.8–70.2% of cases. No pairwise associations between co-infecting pathogens were observed, with the exception of a positive association between *A. phagocytophilum* and *Ehrlichia* sp. infections. The spirochete loads of *B. miyamotoi* were significantly higher than those of *B. burgdorferi* s.l. (mean: 5.2 vs 4.0 log10 genome copies/tick, respectively). Ten isolates of *B. miyamotoi* belonged to the Siberian lineage. *Borrelia burgdorferi* s.l was represented by nine isolates of *B. afzelii, B. bavariensis* and *B. garinii*.

**Conclusions:**

In populations of *I. persulcatus* inhabiting the Selenge and Bulgan provinces of Mongolia, five vector-borne pathogens, i.e. TBEV, *B. burgdorferi* s.l., *B. miyamotoi*, *A. phagocytophilum* and *Ehrlichia* sp., persist independently from each other, with the exception of *A. phagocytophilum* and *Ehrlichia* sp. which seem to share the circulation mode. The discrepancies in *B. burgdorferi* s.l. and *B. miyamotoi* prevalence and spirochete load per tick suggest that different ecological niches are occupied by Lyme disease and relapsing fever agents. High-throughput sequencing allows genetic identification of borreliae species in co-infected ticks.

**Graphical Abstract:**

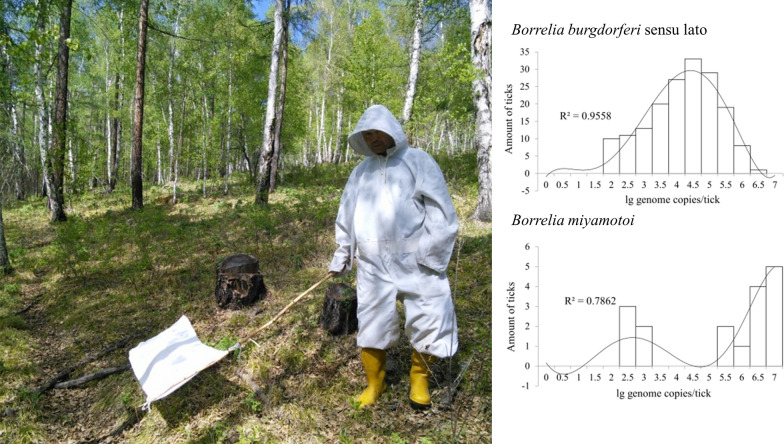

## Background

In Mongolia, tick-borne infections have been recognized as a significant threat to human health since the 1990s. A significant increase in the incidence, geographic range and fatality rates of tick-borne infections has been recorded [1, 2], and the seroprevalence of tick-borne pathogens may reach 15% [3]. About 30.5% of local nomadic herders report a history of tick bites and exposure to some tick-borne pathogens, particularly *Anaplasma* spp. and *Rickettsia* spp., comprising 46.3% of all tick bites [4]. Among the major administrative provinces (hereinafter referred to as aimags) of Mongolia, the northern Selenge and Bulgan aimags are characterized by an especially high risk of tick-borne infections. The landscape of these two adjacent aimags is formed by slopes of the Khentii Ridge and the Selenga River Basin, which provide one of the most humid climate conditions in Mongolia [5]. This results in a wide spread of mixed boreal subalpine forests in the region, with about 33% of land area in Selenge and 29% in Bulgan covered with forests in comparasion to the Mongolian national average of 7.7% [5]. Due to these suitable ecological conditions, the taiga tick *Ixodes persulcatus* (Schulze, 1930) is widely distributed in Selenge and Bulgan aimags where it is the most epidemiologically important vector tick species [6–8]. A number of studies have indicated the presence of such pathogens as tick-borne encephalitis virus (TBEV), *Borrelia burgdorferi* sensu lato (s.l.), *B. miyamotoi*, *Rickettsia sibirica*, *Anaplasma phagocytophilum* and *Ehrlichia muris* in Mongolia ([9], [10], reviewed in [11], [12]). The causative agents of North Asian tick-borne spotted fever, *R. sibirica* and *Rickettsia heilongjiangensis*, are also well recognized human pathogens that are usually associated with *Dermacentor nuttalli* (Olenev, 1928) and *Haemaphysalis concinna* (Koch, 1884) ticks, respectively [13, 14]. However, the infection of *I. persulcatus* with *R. sibirica* and *R. heilongjiangensis* has also been described [15]. In Mongolia, around 4% of *Dermacentor* sp. ticks are infected with *R. sibirica* [12, 16], but no infection of *I. persulcatus* with *R. sibirica* or *R. heilongjiangensis* has been detected so far.

In spite of the high prevalence of tick-borne pathogens in ticks, a relatively small number of disease cases are reported in Mongolia. Thus, the morbidity of Lyme disease in the northern aimags is approximately twofold lower than that in neighboring Russian regions [11]. Also, discrepancies between the number of reported tick bites, symptoms and antibody seroprevalence to tick-borne infections among nomadic Mongolian herders is reported [4]. These discrepancies suggest that ecological and social drivers of tick-borne diseases in Mongolia are still not understood completely. A better characterization of the spatial and temporal distribution of ticks and associated pathogens, long-term surveillance and knowledge of the qualitative characteristics of tick-borne infections are necessary in Mongolia, especially in the northern aimags [4, 11, 12].

The ability of pathogens to infect the same tick lead to multiple co-infections, i.e. simultaneous infection of the same tick specimen with two or more microorganisms. For *I. persulcatus*, co-infections with multiple pathogens have been studied in different parts of the distribution range, including Japan [17–19], Russia [15, 20–22] and Finland [23]. It has been shown that in different geographic locations co-infecting microorganisms can either have no effect on each other, such as TBEV, *B. burgdorferi* s.l. and *A. phagocytophilum* in Western Siberia [24], or there may have positive and negative interactions [22, 25]. In addition, co-infection with some pathogens has an epidemiological importance as it may facilitate the emergence of some zoonoses (e.g. babesiosis in co-infection with he Lyme disease agent) and affect disease severity [25, 26]. For *Borrelia* sp. co-infections are an even more complicated problem due to the significant diversity of these bacteria. Tick-borne borreliae are currently divided into two major groups: the Lyme borreliosis (LB) group and relapsing fever (RF) group [27]. The LB group comprises > 20 species belonging to the *B. burgdorferi* s.l.complex, of which four i.e. *B. burgdorferi* sensu stricto (s.s.), *B. afzelii*, *B. garinii* and *B. bavariensis* are recognized as human pathogens [28]. The RF group is mainly associated with soft (argasid) ticks, although one species, *B. miyamotoi*, is associated with hard (ixodid) ticks and is widely spread across the Northern hemisphere [29]. The regional populations of this pathogen are highly clonal and cluster into three evolutionary lineages designated as Siberian, American and European [30]. While *B. burgdorferi* s.l. and *B. miyamotoi* can infect the same range of tick and vertebrate hosts, co-infections with these pathogens are usual in ticks [30–32]. In Mongolia, the LB group is represented by the genetically diverse *B. afzelii*, *B. garinii* and *B. bavariensis* isolates [33, 34], whereas all known isolates of *B. miyamotoi* are very similar and belong to the Siberian lineage [18]. However, due to high genetic similarity of *B. garinii*, *B. afzelii*, *B. bavariensis* and *B. miyamotoi*, their molecular identification and analysis in co-infected samples is complicated by mixed DNA/RNA templates, especially when the conserved genome regions are investigated [18]. Generally, the information on co-infections of *I. persulcatus* with different pathogens in tick populations in Mongolia is insufficient.

To better understand the ecology of tick-borne infections in Mongolia, we evaluated the contingency of several epidemiologically important infections in the Selenge and Bulgan aimags using co-infection study, quantitatively characterized the infection with *Borrelia* sp. in questing *I. persulcatus* ticks using quantitative PCR (qPCR) and assessed the genetic diversity of *Borrelia* spp. in five ticks infected with *B. miyamotoi* and six ticks co-infected with *B. miyamotoi* and *B. burgdorferi* s.l. using the high-throughput sequencing approach.

## Methods

### Tick collection and processing

**Fig. 1:**
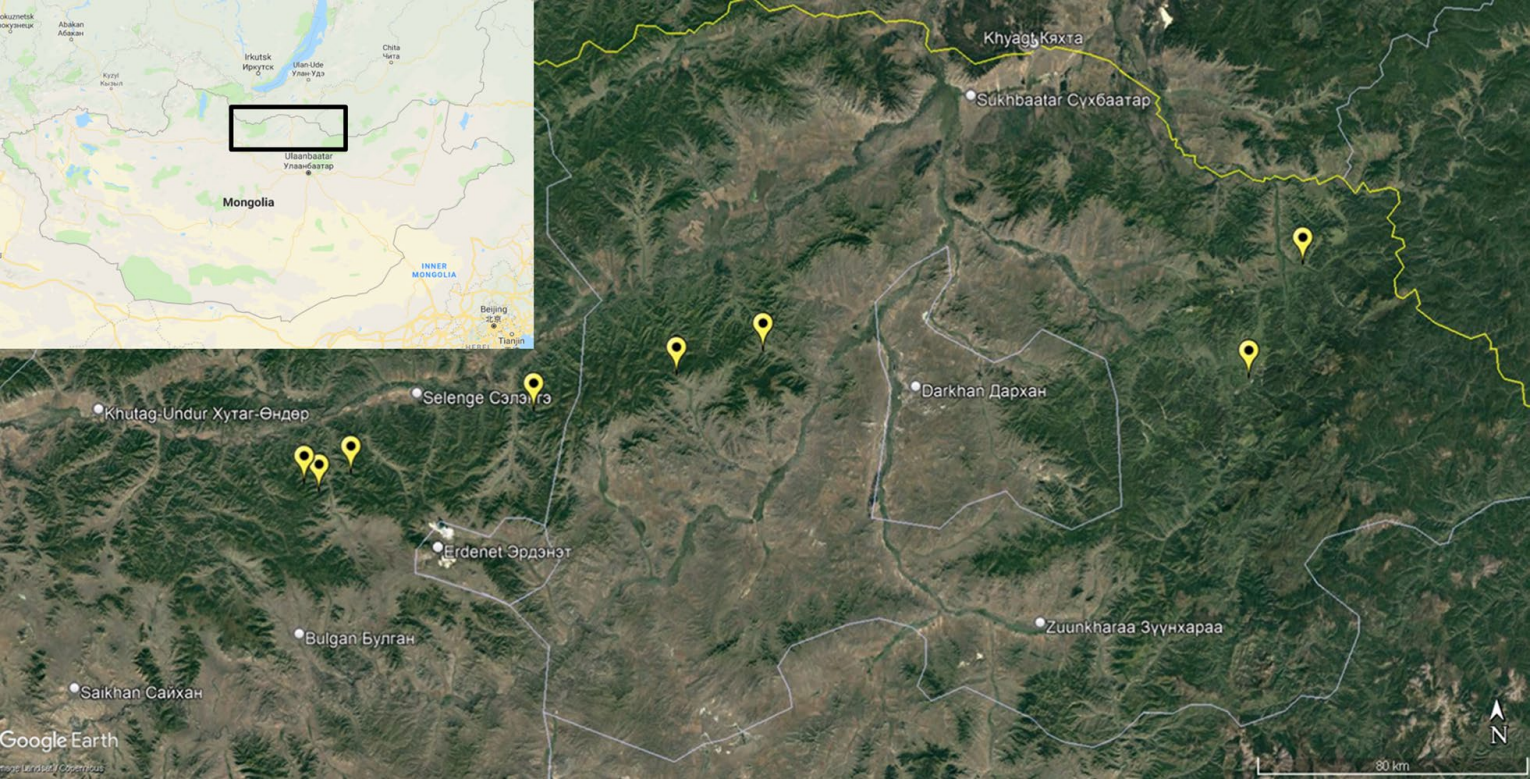
Sampling sites (yellow marks) where questing *Ixodes persulcatus* were collected, in the Selenge and Bulgan aimags of Mongolia. The mapping of sampling sites and the image generation were performed using Google EarthPro 7.3 software (accessed September 2019)

Questing adult *I. persulcatus* ticks were collected from vegetation using flannel flags at eight sampling sites in the Selenge and Bulgan aimags of Mongolia between 30 May 2019 and 2 February 2019. Geographical coordinates of the collection sites were determined using a “Garmin 60CSx” GPS device (Garmin Ltd., Olathe, KS, USA) at the beginning of the tick collection routes and mapped using Google Earth Pro 7.3 software (Fig. 1). Ticks were delivered to the laboratory alive and processed immediately. The ticks were first identified to the species level using a morphological key guide [35]. Afterwards, individual ticks were washed twice in 70% alcohol, once in sterile phosphate buffered saline (PBS, pH = 7.4), crushed with a sterile RNase/DNase-free pestle and resuspended in 100 μl of PBS.

### Extraction of nucleic acids

Total RNA/DNA was isolated from an entire tick suspension using the RealBest Extraction100 kit (VektorBest, Novosibirsk, Russia) according to the manufacturer’s instructions. The final DNA/RNA sample was dissolved in 200 μl of the elution buffer. To prevent any carryover of amplified PCR fragments, tick processing, extraction of nucleic acids from ticks, reverse transcription and preparation of the PCR reaction, and PCR and subsequent electrophoresis were conducted in four separate rooms.

### Reverse transcription, PCR and qPCR

The RNA of TBEV was transcribed with Superscript IV reverse transcriptase (Invitrogen™, Thermo Fisher Scientific, Waltham, MA, USA) and random hexanucleotides according to the manufacturer’s instruction. The primers and probes used in this work are listed in Table 1. Real-time PCR was performed according to the protocol published by Schwaiger and Casinotti [36] with minor modifications. Briefly, the reaction was carried out in a volume of 25 μl that contained 1 U of Taq polymerase HSTaq (Eurogen, Moscow, Russian Federation), 2.5 μl of an optimized reaction buffer (Eurogen), 0.25 mM of each dNTP, the primers F-TBE1 and R-TBE1 at concentration of 50 and 300 nM, respectively, and the probe TBE-WT at concentration of 200 nM (Sintol, Moscow, Russian Federation). The cycling program included preheating at 95 °C for 10 min, followed by 45 cycles of 95 °C for 15 s and 60 °C for 1 min. The results were recorded on the FAM channel at 60 °C.

**Table 1:** Primers and probes

Organism	Primer	Sequence	Target	References
Tick-borne encephalitis virus	F-TBE1	5′-GGGCGGTTCTTGTTCTCC-3′	Primers, 5′ untranslated region	[28]
	R-TBE1	5′-ACACATCACCTCCTTGTCAGACT-3′		
	TBE-WT	5′-FAM-TGAGCCACCATCACCCAGACACA-RTQ1-3′	Probe, 5′ untranslated region	
*Borrelia* sp.	BspF-16s	5′-GCTGTAAACGATGCACACTTGGT-3′	Primers,* 16S* rRNA gene of* B. *sp.	[29, 30]
	BspR-16s	5′-GGCGGCACACTTAACACGTTAG-3′		
	FAM-LD	5′-FAM-TTCGGTACTAACTTTTAGTTAA-MGB-3′	Probe,* 16S* rRNA gene of* B. burgdorferi* s.l	
	VIC-RF	5′-VIC-CGGTACTAACCTTTCGATTA-MGB-3′	Probe,* 16S* rRNA gene of* B. miyamoto*i	
	Bfla-PAD	5′-GATCARGCWCAAYATAACCAWATGCA-3’	Primers, flagellin B gene of* Borrelia* sp.	[31]
	Bfla-PDU	5′-AGATTCAAGTCTGTTTTGGAAAGC-3’		

*rRNA* Ribosomal RNA

Taqman quantitative real-time PCR (qPCR) targeting the* 16S* ribosomal RNA (rRNA) gene of *Borrelia* sp. was used to detect, differentiate and quantify *B. burgdorferi* s.l. and *B. miyamotoi* as described previously [29, 30], with minor modifications. The standard DNA samples of *B. miyamotoi* and *B. burgdorferi* s.l. were prepared according to Takano et al. [30]. The quantity of spirochetes in the specimens was assessed using serial tenfold dilutions of standard DNA samples of corresponding* Borrelia* sp. and expressed as log10 of genome copies per tick. The PCR was performed in a reaction volume of 25 μl containing 1 U of Taq polymerase HSTaq (Eurogen), 2.5 μl of DNA template, primers BspF-16s and BspR-16s at a concentration of 900 nM each and the probes FAM-LD and VIC-RF at a concentration of 200 nM each. The PCR conditions were an initial cycle at 50° C for 2 min, then a cycle at 95 °C for 2 min, followed by 45 cycles of 95 °C for 15 s and 63 °C for 60 s, with reading of the fluorescence.

All samples that were positive for *B. miyamotoi* were subjected to PCR that targeted the flagellin B gene (*flaB*), with primers Bfla-PAD and Bfla-PDU, as described previously [37]. Target PCR fragments of approximately 450 nucleotides were purified by agarose gel electrophoresis and used for high-throughput DNA sequencing (next-generation sequencing [NGS]) library preparation.

The nucleic acids of *A. phagocytophilum*, *E. muris* and/or *E. chaffeensis* (hereafter, *Ehrlichia* sp.) *R. sibirica* and *R. heilongjiangensis* were detected using commercial multiplex real-time PCR kits (VektorBest) according to the manual of the supplier.

All PCR experiments were performed on a CFX Touch T1000 thermal cycler (Bio-Rad Laboratories Inc., Hercules, CA, USA) and processed with Bio-Rad CFX Manager 3.1 software.

### Sequencing

The *flaB* PCR amplicons, purified by agarose gel electrophoresis and the QIAquick Gel Extraction kit (Qiagen, Hilden, Germany), were subjected to NGS analysis on NextSeq 550 sequencer (Illumina Inc., San Diego, CA, USA). Libraries were prepared using the Nextera DNA Flex Library Prep kit, and sequencing was performed using the NextSeq 500/550 Mid Output Kit v2.5 (Illumina) with 300 cycles.

The reads were processed and quality was assessed using the FastQ and FastQC programs implemented in the BaseSpace Sequence Hub (Illumina; https://basespace.illumina.com/dashboard). The sequencing data were then uploaded to the Galaxy web platform, and this public server (https://usegalaxy.org/) was used to analyze the data [38]. The de-novo assembly was performed using the Shovill assembly platform with the Megahit sequence assembler [39]. Resulting contigs were used to generate the consensus sequences, following which the PCR primer sequences were trimmed and obtained sequences were deposited in GenBank under the accession numbers OL580771—OL580777. Sequences were processed and multiple alignments were composed using BioEdit software [40]. The evolutionary distances were computed using the Tamura 3-parameter model [41], which was optimal according to the Bayesian information criterion scores. The evolutionary history was inferred using the maximum likelihood method. The reliability of the trees was evaluated by 1000 bootstrap resampling, and those nodes with bootstrap support > 70% were assumed to be reliable. The phylogenetic analysis was performed using MEGA X sequence analysis software [42].

### Data analysis

The gender ratio and the prevalence of infections was estimated as the proportion of infected ticks and expressed in percentage with 95% confidence intervals (95% CI). The gender rates in different geographic locations and prevalence rates in the tick groups from different geographic locations and genders were compared using the unpaired Chi-square (*χ*^2^) test for proportions.* Borrelia* genome copy number quantification data are presented as mean values with the 95% CI. Differences in quantities of *B. burgdorferi* s.l. and *B. miyamotoi* were analyzed using the unpaired Mann–Whitney test.

To assess the associations between microorganism and frequency of co-infection with any other microorganism, the 2 × 5 contingency table was composed with single-infection and co-infection states of each microorganism determining the binary condition. To assess the associations between TBEV, *B.burgdorferi* s.l*., B. miyamotoi*, *A. phagocytophilum* and *Ehrlichia* sp. infections in ticks, the 2 × 2 contingency tables were constructed for each pair of microorganisms [43], with infected and non-infected states of ticks determining the binary conditions. For each contingency table, the observed values were compared with values expected mathematically under the assumption that the pathogens are distributed in tick populations by chance only. The *χ*^2^ test was used for this comparison, using the Yates correction for tables containing cells with ≤ 5 counts.

The statistical analyses were performed using MS Excel (Microsoft Corp., Redmond, WA, USA), the MaxStat Light free statistical program and R version 4.0.2 (R Foundation, Vienna, Austria) software. Significance was set at 0.95; all significance tests were two-tailed. The differences were considered statistically significant when* P*-values were below 0.05.

## Results

### Tick population

In total, 138 and 208 *I. persulcatus* ticks were sampled from the Bulgan aimag and Selenge aimag, respectively. The proportion of females was 58% (*n* = 80, 95% CI: 49.2–66.2) and 56% (*n* = 114, 95% CI: 48.8–62.8), respectively, and there was no significant difference in gender ratio between geographic locations (*χ*^2^ = 0.07357, *df* = 1, *P* = 0.7862). Four nymphs (1.9%) were collected in the Selenge aimag only.

### Prevalence of infections

Neither *R. sibirica* nor *R. heilongjiangensis* were detected in the entire collected sample and, therefore, these bacteria were not analyzed further. The prevalence of other infections is summarized for each infection separately in Table 2.

**Table 2:** Prevalence of infections in *Ixodes persulcatus* ticks from the Bulgan and Selenge aimags, Mongolia

Collections	Number of ticks studied	Ticks not infected,* n* (%, 95% CI)	Ticks with TBEV,* n* (%, 95% CI)	Ticks with B.b.s.l.,* n* (%, 95% CI)	Ticks with B.m.,* n* (%, 95% CI)	Ticks with A.ph.,* n* (%, 95% CI)	Ticks with E.sp.,* n* (%, 95% CI)
*Bulgan aimag*							
Females	80	28 (35, 25–47)	1 (1.2, 0.06–8)	39 (48.7, 38–60)	5 (6.2, 2.3–15)	3 (3.7, 1–11)*	16 (20, 12–31)
Males	58	19 (32.8, 21–46)	2 (3.4, 0.6–13)	29 (50, 38–62)	3 (5.2, 1.3–15)	9 (15.5, 8–30)*	12 (20.7, 12–34)
Bulgan subtotal	138	47 (34.1, 26–43)	3 (2.2, 0.6–7)	68 (49.3, 41–58)	8 (5.8, 2.7–11)	12 (8.7, 5–15)**	28 (20.3, 14–28)
*Selenge aimag*							
Females	114	37 (32.5, 24–42)	3 (2.6, 0.7–8)	55 (48.2, 39–58)	6 (5.3, 2–12)	25 (21.9, 15–31)	13 (11.4, 6.5–19)
Males	90	36 (40, 30–51)	0 (0, 0–5)	47 (52.2, 41–63)	3 (3.3, 0.9–10)	10 (11.1, 6–20)	14 (15.6, 9–25)
Nymphs	4	3 (75)	0	1 (25)	0	0	1 (25)
Selenge subtotal	208	77 (37, 31–44)	3 (1.4, 0.4–5)	103 (49.5, 43–56)	9 (4.3, 2–8)	35 (16.8, 12–23)**	28 (13.5, 9–19)
Total	346	124 (35.8, 31–41)	6 (1.7, 0.7–4)	171 (49.4, 44–55)	17 (4.9, 3 – 8)	47 (13.6, 10–18)	56 (16.2, 12.5–20.6)

*CI* Confidence interval, *TBEV* tick borne encephalitis virus, *B.b.s.l.* *Borrelia burgdorferi* sensu lato, *B.m.* *Borrelia miyamotoi*, *A.ph.* *Anaplasma phagocytophilum*, *E.sp.* *Ehrlichia* spp.

^*^Prevalence of infection is significantly different between male and female groups (*P* < 0.05)

^**^Prevalence of infection is significantly different between Bulgan and Selenge aimags (*P* < 0.05)

There were no significant differences in the prevalence of infections between different geographic locations or between the groups of male and female ticks, with the exception of *A. phagocytophilum*, which was more prevalent in ticks from the Selenge aimag than in those from the Bulgan aimag (16.8% vs 8.7%, respectively; *χ*^2^ = 4.673, *df* = 1, *P* = 0.0306). This difference was probably due to lower rate of anaplasma infection among females in the Bulgan aimag, and was significant both for females from the Selenge aimag (3.7% vs 21.9%; *χ*^2^ = 11.152, *df* = 1, *P* = 0.0008) and males from the Bulgan aimag (3.7% vs 15.5%; *χ*^2^ = 4.476, *df* = 1, *P* = 0.0344).

Based on these results we assumed that ticks in both the Selenge and Bulgan samples belong to the same general population. We therefore combined the data for further analyses. In the combined sample, the most prevalent pathogen was the *B. burgdorferi* s.l., which was detected in 49% of ticks, followed by *Ehrlichia* sp. (16.0%), *A. phagocytophilum* (13.5%), *B. miyamotoi* (4.9%) and TBEV (1.7%). In total, 64.2% of ticks were infected with at least one pathogen (Table 2).

### Co-infection rate

The data on co-infection by tick-borne pathogens are summarized in Table 3. The majority of infected ticks (70.7%) harbored a single pathogen; however, only *B. burgdorferi* s.l. occurred as single infection (116 ticks), and at an infection rate that was higher than statistically expected (90 ticks). Indeed, 67.0% of all the ticks infected with *B. burgdorferi* s.l. were not infected with any other pathogen. Other pathogens occurred as single infections in 29.8–41.2% of ticks (Table 3), which was less than statistically expected for TBEV (observed single infections vs. single infections: 2 vs. 3 ticks), *B. miyamotoi* (7 vs 9), *A. phagocytophilum* (14 vs 25) and *Ehrlichia* sp. (18 vs 30). Observed correlations were statistically significant (*χ*^2^ = 36.928, *df* = 4, *P* = 0.0001).

**Table 3:** States and prevalence of co-infections of five microorganisms in natural population of *I. persulcatus* (*n* = 346)

Infecting pathogen	Number of ticks positive	Number of ticks with single infection (%, 95% CI)	Number of ticks with co-infections (%, 95% CI)	Co-infecting pathogens					
				TBEV,* n* (%, 95% CI)	B.b.s.l.,* n* (%, 95% CI)	B.m.,* n* (%, 95% CI)	A.ph.,* n* (%, 95% CI)	E.sp.,* n *(%, 95% CI)	> 2,* n* (%, 95% CI)^a^
TBEV	6	2 (33.3)	4 (66.7)		2 (50)	0 (0)	0 (0)	0 (0)	2 (50)
B.b.s.l.	171	116 (67.8, 60–75)	55 (32.2, 25–40)	2 (3.6, 0.6–14)	–	6 (10.9, 4.5–23)	17 (30.9, 20–45)	20 (36.4, 24–50)	10 (18.2, 10–31)
B.m.	17	7 (41.2, 19–67)	10 (58.8, 33.5–81)	0 (0)	6 (60)	–	1 (10)	3 (30)	0 (0)
A.ph.	47	14 (29.8, 18–45)	33 (70.2, 55–82)	0 (0)	17 (51.5, 34–69)	1 (3, 1.6–18)	–	6 (18.2, 8–36)	9 (27.3, 14–46)
E.sp.	56	18 (32.1, 21–46)	38 (67.9, 54–79)	0 (0)	20 (52.6, 36–69)	3 (7.9, 2–23)	6 (15.8, 6, 6–32)	-	9 (23.7, 12–41)

^a^A total of 10 triple infections were detected: TBEV + B.b.s.l. + A.ph. (1 tick); TBEV + B.b.s.l. + E.sp. (1 tick); B.b.s.l. + A.ph. + E.sp. (8 ticks)

Among the co-infected ticks, the most prevalent combinations were *B. burgdorferi* s.l./*Ehrlichia* sp. and *B. burgdorferi* s.l./*A. phagocytophilum*; these combinations were observed in 30.8% and 26.2% of all co-infected ticks, respectively. The rarest combination was co-infection with *B. miyamotoi* and *A. phagocytophilum*, which was detected in 1.5% of co-infections. Notably, there were no cases of multiple (> 2 pathogens) co-infections involving *B. miyamotoi*. Also, of all *B. burgdorferi* s.l.-positive ticks, six (9.2%) were co-infected with *B. miyamotoi*, and the total prevalence of *B. burgdorferi* s.l. + *B. miyamotoi* co-infection in the tick population was 1.7%. No other infections were detected in ticks simultaneously infected with *B. burgdorferi* s.l. and *B. miyamotoi*. The only significant positive association between pairs of infections was co-infection with *A. phagocytophilum* and *Ehrlichia* sp.; this co-infection was detected in 14 ticks, whereas only 7.6 cases were statistically expected if these pathogens were distributed in tick populations by chance only (*χ*^2^ = 6.303, *df* = 1, *P* = 0.0121). Other infections, including every combination that involved *B. burgdorferi* s.l. and *B. miyamotoi*, were not associated with each other, suggesting independent modes of transmission and/or different ecological niches.

### Quantification of *B. miyamotoi* and *B. burgdorferi* s.l.

In *I. persulcatus* ticks, the mean concentration (± 95% CI) of *B. miyamotoi* was 5.2 ± 0.9 log10 genome copies/tick; in comparison, the mean concentration of *B. burgdorferi* s.l. was 4.0 ± 0.2 log10 genome copies/tick. This difference was statistically significant (difference between median values: 2.0 log10 genome copies/tick; *U* = 2110.5, *P* = 0.0021), indicating that the mean load of *B. miyamotoi* was approximately 28-fold higher than that of *B. burgdorferi* s.l. To verify that these results are not affected by artifacts of the qPCR test (e.g. by non-specific probe hydrolysis at the late stages of PCR), we compared those ticks with a *Borrelia* spp. load of > 10 genome copies per reaction. We found that the mean spirochete load was 6.25 ± 0.4 and 4.3 ± 0.4 log10 genome copies/tick for *B. miyamotoi* and *B. burgdorferi* s.l., respectively (difference between median values: 2.05 log10 genome copies/tick; *U* = 1677, *P* = 0.0001), thereby supporting the significance of an approximately 30-fold higher mean spirochete load of *B. miyamotoi* in comparison to *B. burgdorferi* s.l.

In five co-infected ticks, the load of *B. miyamotoi* was approximately 10- to 50-fold higher than that of *B. burgdorferi* s.l., with mean quantities of 4.5 ± 0.8 and 3.4 ± 0.4 log10 genome copies per tick, respectively (difference in median values: 1.363 log10 genome copies/tick; *U* = 23, *P* = 0.028). In the sixth co-infected tick, the concentration of *B. miyamotoi* was lower than that of the *B. burgdorferi* s.l.; however this quantification of *B. miyamotoi* was not confirmed by *flaB* sequencing (Table 4).

**Table 4:** Borreliae quantification and species identification in co-infected *Ixodes persulcatus* ticks

Tick ID	Representative *flaB* sequence accession no.	Bacterial genome load (log10 copies/tick)		De-novo sequence assembly coverage (average reads per base [Megahit])			
		B.m.	B.b.s.l.	B.m.	B.a.	B.b.	B.g.
Mng_A19-2	*B. miyamotoi* OL580771	6.9	nd	655	nd	nd	nd
Mng_A19-7	*B. afzelii* OL580772, *B. bavariensis* OL580774	6.7	5.6	625	43	35	nd
Mng_A19-58	*B. bavariensis* OL580775	6.9	5.5	1001	nd	68	nd
Mng_B19-11	*B. bavariensis* OL580776	5.3	5.3	227	nd	946	nd
Mng_B19-16	*B. afzelii* OL580773	2.5	6.5	nd	407	nd	nd
Mng_B19-54	Identical to OL580771	5.6	nd	865	nd	nd	nd
Mng_C19-12	Identical to OL580771	6.1	nd	896	nd	nd	nd
Mng_C19-45	Identical to OL580771, OL580772 and OL580775	6.4	5.1	488	97	5	nd
Mng_C19-80	Identical to OL580771	6.6	nd	424	nd	nd	nd
Mng_C19-104	Identical to OL580771	6.5	nd	16591	nd	nd	nd
Mng_C19-111	*B. garinii* OL580777 Identical to OL580771 and OL580772	6.6	4.8	591	100	nd	43
Mean ± 95% CI		6.0 ± 0.9	5.5 ± 0.6	2236 ± 3612	162 ± 264	264 ± 725	43

*B.a.* *B. afzelii*, *B.b.* *B. bavariensis*, *B.g. B. garinii, nd* not detected

**Fig. 2:**
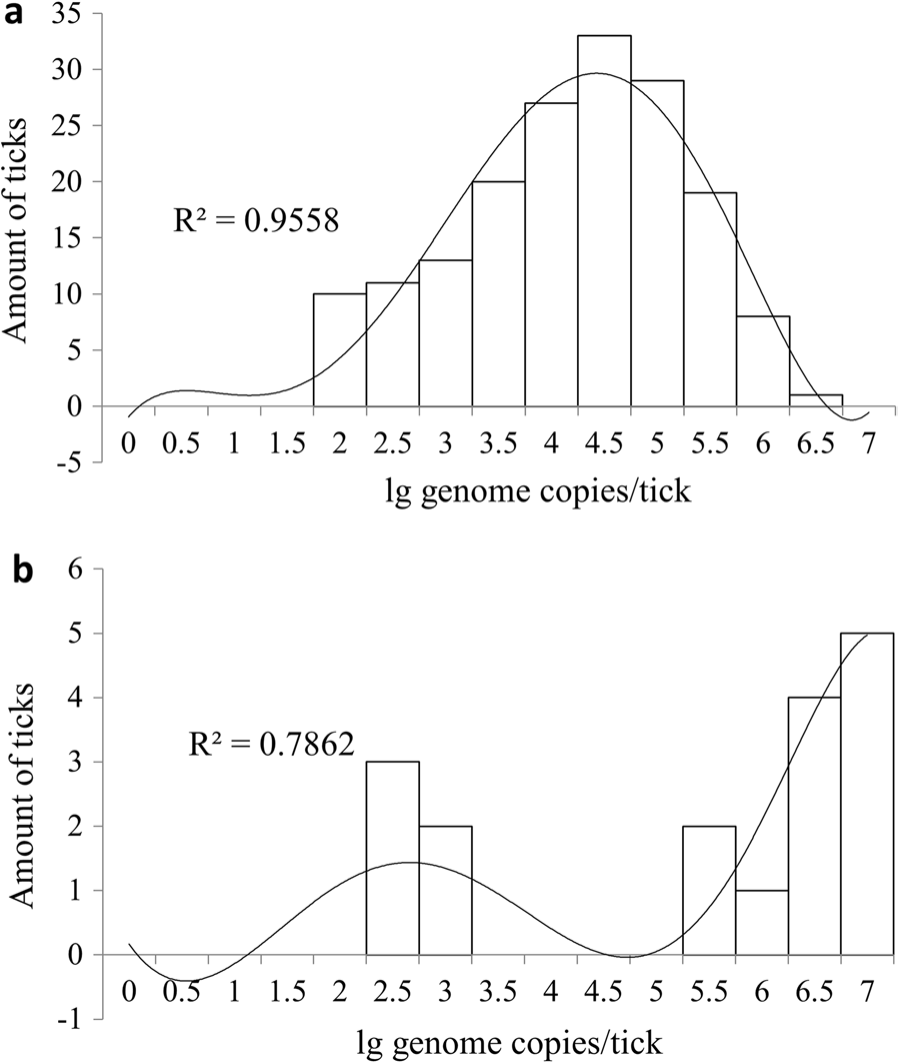
Histograms of the frequency distribution of spirochete loads of *Borrelia burgdorferi* sensu lato,* n* = 171 (**a**) and *Borrelia miyamotoi*,* n* = 17 (**b**) in infected *Ixodes persulcatus* ticks. Trend lines were constructed using a polynomial regression model

The distribution of the log-transformed spirochete amounts in the infected ticks significantly differed between *B. burgdorferi* s.l. and *B. miyamotoi*. The amounts of *B. burgdorferi* s.l. were normally distributed in general, with a slight tail to the low values (Fig. 2a). However, the distribution of amounts of *B. miyamotoi* had a bimodal shape: the load of the first group of ticks was between 2.5 and 3 log10 genome copies/tick, and that of the second group of ticks was– between 5.5 and 7 log10 genome copies/tick (Fig. 2b).

### Genotyping of *Borrelia* sp*.* in co-infected samples

All 17 samples infected with *B. miyamotoi* were subjected to PCR targeting *flaB* gene fragment. Of these, five failed to produce any PCR product; four of these *flaB*-negative samples contained low amounts of *B. miyamotoi* DNA (2.9–7.5 genome copies/tick), while the fifth sample had a high bacterial load of 28250 *B. miyamotoi* genome copies/tick according to* 16S* rRNA qPCR. Of the other 12 samples, one did not produce sufficient amount of PCR product to perform further research. The remaining 11 amplicons, including six co-infected with *B. burgdorferi* s.l., were successfully analyzed using NGS. The primer sequences were trimmed from the final gene fragments, and 10 sequences of *B. miyamotoi*, five sequences of *B. bavariensis* and four sequences of *B. afzelii* 397-bp *flaB* gene fragments were assembled (Table 4).

Five samples were infected solely with *B. miyamotoi*, which was consistent with the qPCR data. Co-infected sample Mng_B19-16 did not contain any sequences of *B. miyamotoi flaB*, and only the *flaB* fragment of *B. afzelii* was detected in this sample. Of the remaining five co-infected samples, two were double infections with *B. miyamotoi* and *B. bavariensis*, two were triple infections with *B. miyamotoi*, *B. bavariensis* and *B. afzelii* and one was a triple infection with *B. miyamotoi*, *B. garinii* and *B. afzelii*. In total, of the 11 analyzed samples, five were infected with *B. miyamotoi* only, one was infected with *B. afzelii* only, two were co-infected with two pathogens and three were co-infected with three* Borrelia* species (Table 4). The Megahit assembly coverage varied from 5 to 16,591 reads per base and, on average, comprised 2236 reads per base for *B. miyamotoi*, 264 reads per base for *B. bavariensis*, 162 reads per base for *B. afzelii* and 43 reads per base for *B. garinii* (Table 4).

**Fig. 3:**
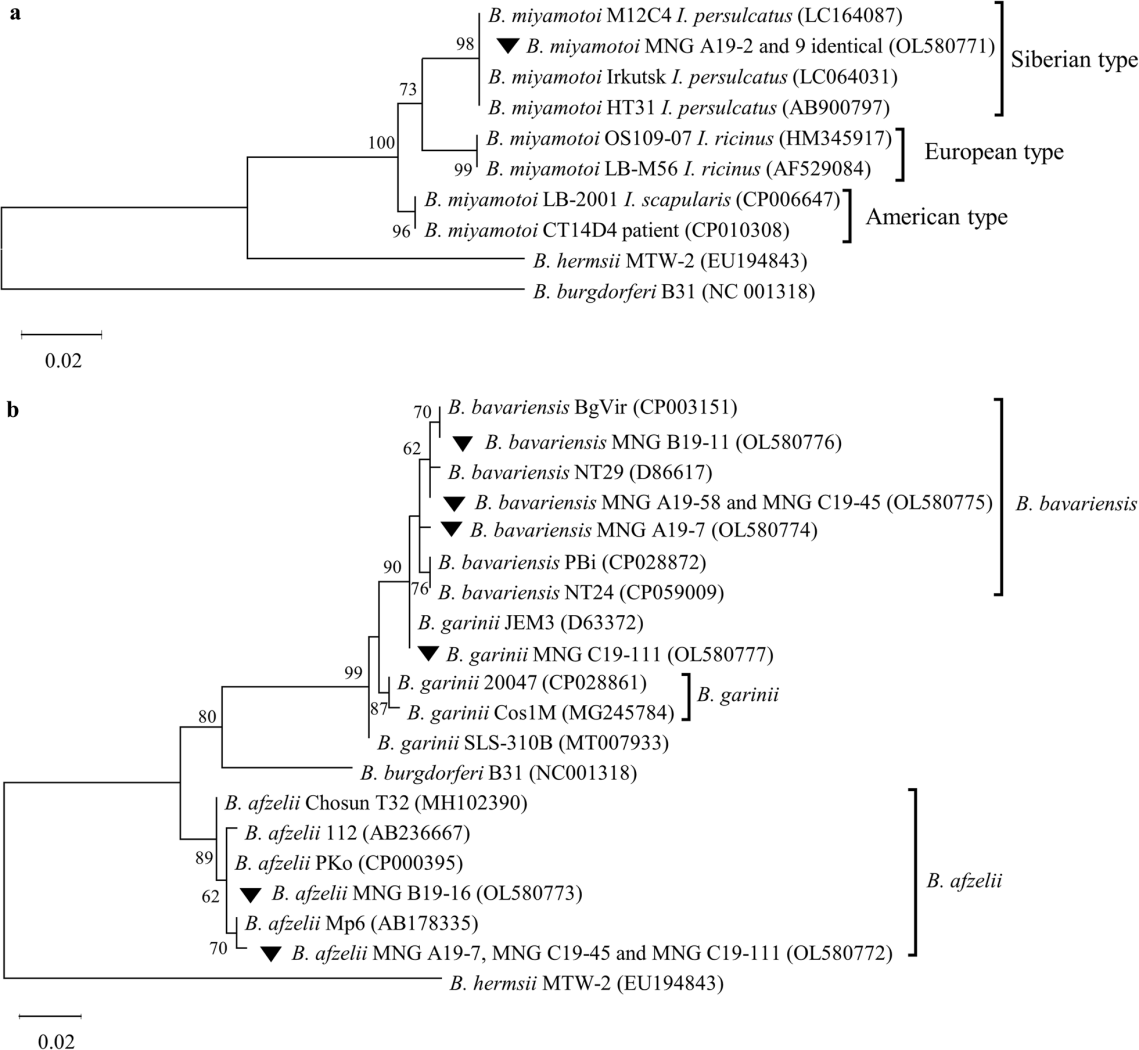
Phylogenetic relationships of *B. miyamotoi* (**a**) and *B. burgdorferi* sensu lato (**b**) co-infecting *I. persulcatus* ticks. The isolates obtained in this work are indicated by inverted triangles. The evolutionary history was inferred using the maximum likelihood method. The evolutionary distances were computed using the Tamura 3-parameter model TN92. The scale indicates the number of base substitutions per site. The bootstrap support (in percentage, from 1000 replicates) is shown next to the branches; bootstrap values < 70% are hidden. Evolutionary analyses were conducted in MEGA X software

All samples of *B. miyamotoi*, both single- and co-infecting *I. persulcatus*, had the identical *flaB* sequences belonging to the Siberian lineage. All of these sequences were identical to the reference strain *B. miyamotoi* HT31, isolated from *I. persulcatus*, as well as with previously described isolates from the Irkutsk Region and Mongolia (Fig. 3a).

The *B. bavariensis* nucleotide sequences could be divided into three distinct genotypes. Sample Bb_Mng_B19-11 was closely related to the BgVir strain from the Tomsk Region; two identical samples, Bb_Mng_A19-58 and Bb_Mng_C19-45, clustered with the well-described strain NT29; and Bb_Mng_A19-7 clustered with the reference strain of *B. bavariensis* PBi (Fig. 3b). At the amino acid level, all studied samples were identical to *B. garinii* 20047 and *B. bavariensis* NT29, but not to the *B. bavariensis* PBi that contained mutation A203S (the residue numbering of *B. burgdorferi* s.s. B31, accession NC_001318).

The sample Bg_Mng_C19-111 was identical to *B. garinii* strain JEM3 whose *flaB* nucleotide sequence was unique in the NCBI BLAST database. The amino acid sequence of Bg_Mng_C19-111 was identical to both *B. garinii* 20047 and *B. bavariensis* NT29.

The *B. afzelii* samples were represented by two genotypes. Three samples (i.e. Ba_Mng_A19-7, Ba_Mng_C19-45 and Ba_Mng_C19-111) were most closely related to the strain Mp6 that was isolated from *I. persulcatus* in the European part of Russia [44]. Sample Ba_Mng_B19-16 was identical to the *B. afzelii* PKo strain isolated from the skin of patient in Germany [45], as well as to a number of Siberian and European *B. afzelii* isolates from different sources. At the amino acid level, samples Ba_Mng_A19-7, Ba_Mng_C19-45 and Ba_Mng_C19-11 had the unique mutation A209S in comparison to the *B. afzelii* PKo strain (in the residue numbering of NC_001318). No identical mutations were found in other *B. afzelii* isolates in the NCBI database using BLASTp service.

## Discussion

### The prevalence of infection

The overall prevalence of TBEV in *I. persulcatus* in 2019 comprised 1.7% (95% CI: 0.7–4.0), with no significant difference between the Selenge (1.4%, 95% CI: 0.4–5.0) and Bulgan (2.2%, 95% CI: 0.6–7.0) aimags. TBEV prevalence in previous studies was mainly reported for the Selenge aimag and recorded in different years as being 5.5% [46], 1.6% [47], 1.3% [48] and 1.9% [49], with an average estimation of 3.2% [11]. In the Bulgan aimag, the prevalence of TBEV has been reported to vary from 1.5% [11] to 4.4% [46]. Thus, our results, in general, correspond to the most recent evaluations. A slight trend towards a decrease in the overall prevalence of TBEV in *I. persulcatus* may be assumed. However, a larger sample of ticks and a longer observation period are necessary to precisely evaluate the dynamics of TBEV infection in Mongolian populations of *I. persulcatus*.

The prevalence of *B. burgdorferi* s.l. in Mongolian populations of *I. persulcatus* in different years was reported to be 32.8–36.1% [9], 22–24.5% [34], 55% [19] and 45% [18]. In 2019, the prevalence of the Lyme disease agent was 49.4%, which is close to the highest values registered during the entire period of our surveillance in Mongolia, indicating the high risk of human cases of Lyme disease.

The relapsing fever agent *B. miyamotoi* from the questing ticks collected in the 2013–2015 periodwas reported in Mongolia in 2017 for the first time [18]. In that period, the prevalence of the infection in *I. persulcatus* was the same in the Selenge, Bulgan and Huvsugul aimags, averaging 4.5% [18]. This value is very similar to our observations of the pathogen 4–6 years later (4.9%). In other populations of *I. persulcatus*, the prevalence of the pathogen varied, being 1.6–2% in Japan [18, 30], 2.6–5% in neighboring Inner Mongolia, China [50, 51], 1.7–2.9% in the Irkutsk Region [37, 52], up to 6.3% in Western Siberia [15], 1.8% in the European parts of Russia [53] and about 4% in Finland [54]. Thus, the mean prevalence of *B. miyamotoi* in Mongolian taiga ticks seems to be relatively high in comparison with other geographic locations, which may increase the risk of relapsing fever disease in the Selenge and Bulgan aimags.

In the Selenge aimag, *A. phagocytophilum* was previously reported in 6.2% of ticks [55], whereas *Ehrlichia muris* was only detected in 0.1–2.5% of *I. persulcatus* ticks [10]. In our samples, these microorganisms were two- to fivefold more prevalent (13.6% vs 16.2%, respectively), which is unusual, not only for Mongolia but also for other geographic locations. There have been several cases of *R. sibirica* isolation from *I. presulcatus* in Western Siberia [56]; these previous results allowed us to assume the vector competence of the taiga tick for this pathogen. In addition, Rar and colleagues, who studied 334 adult ticks of *I. persulcatus* from Western Siberia, reported the prevalence of *R. sibirica* and *R. heilongjiangensis* in those ticks to be 2.4% and 0.3%, respectively [15]. In contrast, we did not observe either of these two rickettsia species in taiga ticks despite having a comparable sample size of 346 specimens. In our opinion, the vector ability of *I. persulcatus* for these bacteria is minimal (if any), whereas the reported cases are sporadic and most likely caused by accidental pathogen “leakage” from sympatric vertebrate and invertebrate reservoir hosts.

The observed variability in prevalence of tick-borne infections at the same locations of Mongolia during the time may be caused not only by natural processes (e.g. if local ecosystems became more suitable for particular pathogen) but by technical biases as well (e.g. by differences in pathogen detection techniques, variable amount of sampling sites, etc.). Further prolonged observations and intense surveillance using unified methodology would help to elucidate the dynamics of tick-borne infections in Mongolian populations of *I. persulcatus*.

### Co-infections

In this work we studied the co-infection of *I. persulcatus* in Mongolia with seven epidemiologically significant pathogens; this is the first report of a study using this approach. However, the study is restricted by our assumption that *B. bavariensis*, *B. afzelii* and *B. garinii* act as a single pathogen of the *B. burgdorferi* s.l. complex. However, we propose that our results in this study case are valid from epidemiological perspective because each of these borrelias is a well-recognized agent of Lyme disease, and no other species from the *B. burgdorferi* s.l. complex has ever been detected in Mongolia or neighboring territories [18, 33, 34, 57, 58].

The abundance of co-infections appeared to be quite high, with about one third of all infected ticks being co-infected with two or more pathogens. The vast majority of co-infections were caused by two pathogens, and no ticks were infected with more than three pathogens. All co-infections involved different combinations of TBEV, *B. burgdorferi* s.l., *A. phagocytophilum* and *Ehrlichia* sp. (most likely *E. muris*). Interestingly, *B. miyamotoi* was present at the same prevalence as TBEV; however, the only co-infection observed for this pathogen was for the pair *B. burgdorferi* s.l./*B. miyamotoi*, with no statistically significant contingency between these spirochetes. In contrast, TBEV was observed in co-infection with *B. burgdorferi* s.l., *A. phagocytophilum* and *Ehrlichia* sp.

In Mongolia, co-infections with varying combinations of tick-borne pathogens have been occasionally reported in previous studies. For the pair *B. burgdorferi* s.l./*A. phagocytophilum*, the prevalence of co-infections was determined to be 4.7% in the Selenge aimag [19]. Co-infection with *B. miyamotoi* and *B. burgdorferi* s.l. was observed in 1.2% of ticks from the Selenge, Bulgan and Huvsgol aimags [18]. In our study, corresponding rates were very similar, being 4.9% and 1.7%, respectively.

The independent distribution of pathogens in ticks suggest differences in vertebrate hosts, mechanisms of transmission and, probably, different tissue tropism in vertebrates and ticks.

### Quantitative characteristics of *Borrelia* sp. in *I. persulcatus*

The quantitative characteristics of *Borrelia* sp. in *I. persulcatus *found in the present study are in very good agreement with previously published data on *I. scapularis* in the USA [29], especially in terms of the almost identical shape of the frequency distribution curves (Fig. 2). However, in our study, the mean spirochete loads in *I. persulcatus* were approximately 10- to 100-fold higher higher for both species. Thus, for *B. burgdorferi* s.l., mean counts in *I. scapularis* versus *I. persulcatus* were 3155 (or 3.5 log10) and 4.0 log10 spirochetes/infected tick, respectively. For *B. miyamotoi*, the difference in spirochete loads was even more notable, comprising 4246 (or 3.6 log10) and 5.9 log10 genome copies, respectively. There are two major considerations that may explain these differences. First, differences in the species and life stage studied: in the present study, we studied *I. persulcatus* adults, while Barbour et al. [29] studied *I. scapularis* nymphs. Both the species of tick and the life stage may affect the amount of spirochetes harbored by an infected tick. Secondly, our DNA extraction protocol differed from that used by Barbour et al. [29], possibly increasing the detection limit. The synergy of these two considerations may also have occurred. It should be noted, however, that under laboratory conditions, the concentration of *B. burgdorferi* s.s. in infected nymphs of *I. scapularis* was found to be 4–5 log10 genome copies/tick [59], which is is very similar to values found in natural populations of *I. persulcatus*.

In contrast, in the study of questing adult *I. persulcatus* in the Far East of Russia, the average load of * B. burgdorferi* s.l. was evaluated to be 5.6 × 10^7^ (or 7.7 log10) genome equivalents/tick [60], which is approximately 1000-fold higher than the values observed in our survey (4.0 log10). The data on *B. miyamotoi* quantification are also contradictive: 9.52 × 10^3^ (or 4.0 log10) versus to 5.2 log10, respectively. This discrepancy, however, could be explained by technical biases. Indeed, Pukhovskaya and colleagues used a different quantification approach that initially had been developed for *B. burgdorferi* s.l. solely [61], and *B. burgdorferi* s.s. B31 was used as universal standard to quantify both *B. burgdorferi* s.l. and *B. miyamotoi* [60]. We used the same approach as Barbour and colleagues [29] and quantified both borrelia species simultaneously in a one-tube multiplex qPCR test using separate sets of DNA copy number standards for *B. burgdorferi* s.l. and *B. miyamotoi*. As well, our quantification results are supported by two independent methods, namely the qPCR assay of the* 16S* rRNA gene and NGS analysis of the *flaB* gene fragment. Therefore, we hypothesize that *B. burgdorferi* s.l. and *B. miyamotoi* co-exist in the ecosystems with *I. persulcatus* as a competent vector using the mechanism of partitioning of ecological niches, similar to that discovered for *B. burgdorferi* s.s. and *B. miyamotoi* in ecosystems with *I. scapularis* being the competent vector [29].

### Genetic diversity of *Borrelia *spp*.* in co-infected ticks

To identify the species and perform phylogenetic analysis of *Borrelia spp*. in six ticks co-infected with *B. burdorferi* s.l. and *B. miyamotoi* and in five ticks with single infection of *B. miyamotoi*, we used the high-throughput massive parallel sequencing of the *flaB* gene fragment. *flaB* is a chromosomal gene encoding flagellin B, a core protein in the spirochete periplasmic flagella [62]. This gene is localized in a chromosomal part of the genome and is highly conserved among different species of the genus *Borrelia*, which makes it a popular target for universal PCR detection systems as it allows the detection and quantification of borrelia infections irrespective of the causative species and plasmid strain [63, 64]. Despite its high level of conservation, there are numerous minor differences in the nucleotide sequences of *flaB*, and these enable both the species of borreliae to be identied as well as reconstruction of the phylogenetic relationships of the borreliae [19, 65, 66].

Using this approach, we successfuly characterized *Borrelia* sp. diversity in six co-infected ticks. It appeared that up to three different species, i.e. *B. miyamotoi*, *B. afzelii* and *B. bavariensis* or *B. garinii*, may infect a single tick simultaneously. The prevalence of triple borrelial infections was quite high, about 1% of the entire *I. persulcatus* population, however no quadruple infection was detected. Previously, only dual co-infections of *B. garinii* with *B. afzelii* (7.8%) or *B. bavariensis* (6.2%) were reported [19].

The Mng_B19-16 sample contained both *B. miyamotoi* and *B. burgdorferi* s.l. DNA according to the qPCR targeting the* 16S* rRNA gene. However, only *B. afzelii* was detected in the sample when the *flaB* amplicon was analyzed. In this sample, the concentration of *B. miyamotoi* was approximately 10,000-fold lower than that of *B. afzelii* (2.5 vs 6.5 log10 copies/tick, respectively), whereas in other co-infected samples the difference in concentrations did not exceed 100-fold (Table 4). Most likely, the less abundant *flaB* template of *B. miyamotoi* failed to compete with the much more abundant *B. afzelii* template. In this case, for successful resolution of closely related pathogens in co-infected ticks, the difference in concentrations of the microorganisms should not exceed 2–3 log10 genome copies per sample. Another explanation could be false-positive signal for *B. miyamotoi* during qPCR targeting the * 16S* rRNA gene due to, for example, non-specific Taqman probe binding and hydrolysis on the template of *B. afzelii* or some other bacteria in that particular tick. In this case, every sample with a low concentration of *B. miyamotoi* should be assumed as suspicious. Notably, every single-infected sample with a very low (below 10 copies per tick) concentration of *B. miyamotoi* failed to produce the *flaB* PCR fragment. To resolve this issue, the detailed study of ticks with low concentrations of *Borrelia* sp. is necessary.

There was no diversity in *B. miyamotoi* detected in Mongolia. All characterized isolates were highly homogeneous and belonged to the Siberian lineage that is associated with *I. persulcatus* and widely distributed from Japan to Europe [18, 67]. In contrast, the remarkable diversity of Lyme disease agents was described. Thus, it had been shown previously that 26.3% of *B. burgdorferi* s.l. belong to *B. garinii* 20047, followed by 7.8% of *B. afzelii* and 7.0% of *B. garinii* NT29 type (nowadays *B. bavariensis*) [19]. In another study of 91 borreliae-positive ticks, 62% were infected with the *B. bavariensis*, 13% were infected with *B. afzelii* and 15% were co-infected with both species [34]. Generally, the genetic diversity of *Borrelia* sp. in co-infected samples in our study is rather high and corresponds to previously published data [34]. No strict geographic associations in the phylogeny of *Borrelia* sp. were detected for studied isolates from Mongolia, which could be explained by an intense exchange of the isolates between different populations of *I. persulcatus* and with closely related *I. ricinus* ticks. These results also characterize the NGS as a powerful tool that is very effective in studies of tick-borne co-infections.

## Conclusion

At least five vector-borne pathogens, i.e. TBEV, *B. burgdorferi* s.l., *B. miyamotoi*, *A. phagocytophilum* and *Ehrlichia* sp. (most likely, *E. muris*), simultaneously circulate among *I. persulcatus* ticks in the Selenge and Bulgan aimags of Mongolia, and > 64% of ticks are infected with at least one pathogen. The analysis of contingency tables of infections indicates that these microorganisms persist independently of each other and, probably, occupy different ecological niches. The only exception is the pair *A. phagocytophilum* and *Ehrlichia* sp., which occur significantly more often as co-infection than expected for independent distribution, suggesting a common mode of transmission, shared range of vertebrate hosts and tissue tropism. Among the *I. persulcatus* ticks, *B. burgdorferi* s.l. had a tenfold higher prevalence of infection than *B. miyamotoi*, but the spirochete loads of *B. burgdorferi* s.l. in infected ticks were, vice versa, 28- to 30-fold lower than concentration of *B. miyamotoi*. From the healthcare perspective, this result could mean that in spite of a lower abundance of relapsing fever agent, the risk of infection with this pathogen is similar to that of Lyme disease due to the higher initial dose of pathogen in each case of infection. The analysis of the *flaB* gene fragment of *Borrelia* sp. in five single-infected and six co-infected samples revealed that all 10 *B. miyamotoi* isolates belong to the Siberian evolutionary lineage. The *Borrelia burgdorferi* s.l. was represented by *B. afzelii* (four isolates), *B. bavariensis* (four isolates) and *B. garinii* (one isolate). The most diverse group was *B. bavariensis*, consisting of three genotypes, and *B. afzelii*, divided into two genotypes; in contrast, *B. garinii* was represented by single isolate only. A unique amino acid mutation of alanine to serine was discovered in residue 209 of the *flaB* gene of Mongolian isolates of *B. afzelii*. Introduction of quantitative analysis of the kinetics of tick-borne infections in vectors, vertebrate hosts and human patients would enhance our understanding of the persistence of these pathogens in nature and help to improve the preventive measures, diagnostics and treatment of tick-borne diseases.

## Data Availability

The data generated in this study are available from the corresponding author upon request.
